# Genetic basis of rotator cuff injury: a systematic review

**DOI:** 10.1186/s12881-019-0883-y

**Published:** 2019-09-02

**Authors:** Umile Giuseppe Longo, Vincenzo Candela, Alessandra Berton, Giuseppe Salvatore, Andrea Guarnieri, Joseph DeAngelis, Ara Nazarian, Vincenzo Denaro

**Affiliations:** 1 Department of Orthopaedic and Trauma Surgery, Campus Bio-Medico University, Via Alvaro del Portillo, 200, Trigoria, 00128 Rome, Italy; 2 Carl J. Shapiro Department of Orthopaedic Surgery and Center for Advanced Orthopaedic Studies, Beth Israel Deaconess Medical Center and Harvard Medical School, Boston, MA USA

**Keywords:** Rotator cuff, Gene, Genetic, Shoulder, Predisposition

## Abstract

**Background:**

Rotator cuff disease is a widespread musculoskeletal pathology and a major cause of shoulder pain. Studies on familial predisposition suggest that genetic plays a role in the pathogenesis of rotator cuff disease. Several genes are responsible for rotator cuff disease. The aim of this study was to perform a systematic review on genetic association between rotator cuff disease and genes variations.

**Methods:**

A systematic review of the literature was performed, in accordance with the PRISMA guidelines. PubMed, Medline, CINAHL, Cochrane, Embase and Google Scholar databases were searched comprehensively using the keywords: “Rotator cuff”, “Gene”, “Genetic”, “Predisposition”, “Single-nucleotide polymorphism” and “Genome-wide association”.

**Results:**

8 studies investigating genes variations associated with rotator cuff tears were included in this review. 6 studies were case-control studies on candidate genes and 2 studies were GWASs. A significant association between SNPs and rotator cuff disease was found for DEFB1, FGFR1, FGFR3, ESRRB, FGF10, MMP-1, TNC, FCRL3, SASH1, SAP30BP, rs71404070 located next to cadherin8. Contradictory results were reported for MMP-3.

**Conclusion:**

Further investigations are warranted to identify complete genetic profiles of rotator cuff disease and to clarify the complex interaction between genes, encoded proteins and environment. This may lead to individualized strategies for prevention and treatment of rotator cuff disease.

**Level of evidence:**

Level IV, Systematic Review.

## Background

Rotator cuff disease is a widespread musculoskeletal pathology and a major cause of shoulder pain [1]. This disabling condition has high prevalence, affecting 30–50% of the population older than 50 years of age [2]. Rotator cuff disease is a common health concern among working populations. The impact of this condition on earnings, missed workdays, and disability payments is relevant [2].

The etiology of rotator cuff disease is multifactorial [2–7] and its pathogenesis is not completely understood. In addition to aging, several factors can contribute to its etiopathogenesis, such as overuse, mechanical impingement, and smoking [3, 5, 8]. Studies on familial predisposition suggest that genetic plays a role in the pathogenesis of rotator cuff disease. Family members of patients with rotator cuff tears have a significantly higher risk of rotator cuff tears than general population [9, 10]. Tashjian et al. determined an increased risk of tears in family members of patients with rotator cuff tears that extends out and beyond third-cousin relationships [11]. Genetic predisposition may play a role also in clinical presentation and progression of rotator cuff tears. Genetically susceptible patients experience symptoms more often [12–15], in fact the relative risk of having a painful tear is 1.44 for siblings of a symptomatic patient [16]. These inheritable characteristics may affect any point of the sensorineural pathway. Moreover, the progression of a tear over a five-year period, is greater in siblings than in controls (tear size increased in 16.1% of siblings, compared with 1.5% of control group) [16].

Several genes are responsible for rotator cuff disease. Genetic susceptibility may affect the ultrastructure of the tendon. Achilles tendinopathy has been associated with polymorphisms of tenascin C and collagen type Va [17]. Similar mechanisms could play a role in the pathogenesis of rotator cuff disease. The genetic basis of this condition may also result from aberrations in the normal cell regulation of apoptosis and tissue regeneration.

The aim of this study was to perform a systematic review on genetic association between rotator cuff disease and candidate genes.

## Methods

A systematic review of the literature was performed in accordance with the Preferred Reporting Items for Systematic Reviews and Meta-Analyses (PRISMA) guidelines with a PRISMA checklist and algorithm.

A comprehensive search of MEDLINE, PubMed, Cochrane, EMBASE, CINAHL, and Google Scholar databases using various combinations of the keywords: “Rotator cuff”, “Gene”, “Genetic”, “Predisposition”, “Genome-wide association”, “Single-nucleotide polymorphism” was performed. Three independent reviewers (U.G.L., V.C., and A.B.) conducted the search separately. All scientific journals were considered, and all relevant studies were analysed.

In order to qualify, an article must have been published in a peer-reviewed journal All articles were initially screened for relevance. The three investigators separately reviewed each abstract and completed a close reading of all articles to minimize selection bias and error. According to the Oxford Centre of Evidence-Based Medicine, only Level I to Level IV articles in English were included in our study.

We included articles that described genetic variations (single-nucleotide polymorphism, SNP) associated with rotator cuff disease. Both case-control studies focused on candidate genes and Genome-wide association studies (GWASs) were included. Studies should clearly describe the criteria used for the diagnosis of rotator cuff disease, genes and SNPs investigated. Missing data relevant to these parameters warranted exclusion from this systematic review. We did not included studies about familial predisposition, genetic variants and gene expression patterns associated with rotator cuff disease and/or healing. Literature reviews, case reports, animal and cadaveric studies, technical notes, letters to the editor, and instructional courses were excluded.

Finally, in order to avoid bias, the selected articles and their references, and the articles excluded from the study were reviewed, evaluated, and discussed by all the authors. All investigators independently extracted the type of study, number of cases and controls, diagnostic criteria of rotator cuff disease, investigated genes, mean age of cases and controls.

### Quality assessment

We used the Coleman Methodology Score to assess the quality of the selected studies (CMS) [18], in which ten criteria are used to render a score ranging from 0 to 100 points (a score of 100 indicating a study that largely avoids chance, various biases, and confounding factors). The final score is defined as excellent (85 to 100 points), good (70 to 84 points), fair (50 to 69 points), and poor (< 50 points).

The subsections of the CMS are based on the subsections of the CONSORT statement outlined for randomized controlled trials, which have been modified to allow for other trial designs. Coleman criteria were subjected to modification in order to make them reproducible for this systematic review. Each study was scored by the three reviewers (U.G.L., V.C. and A.B) independently and in triplicate.

## Results

**Fig. 1:**
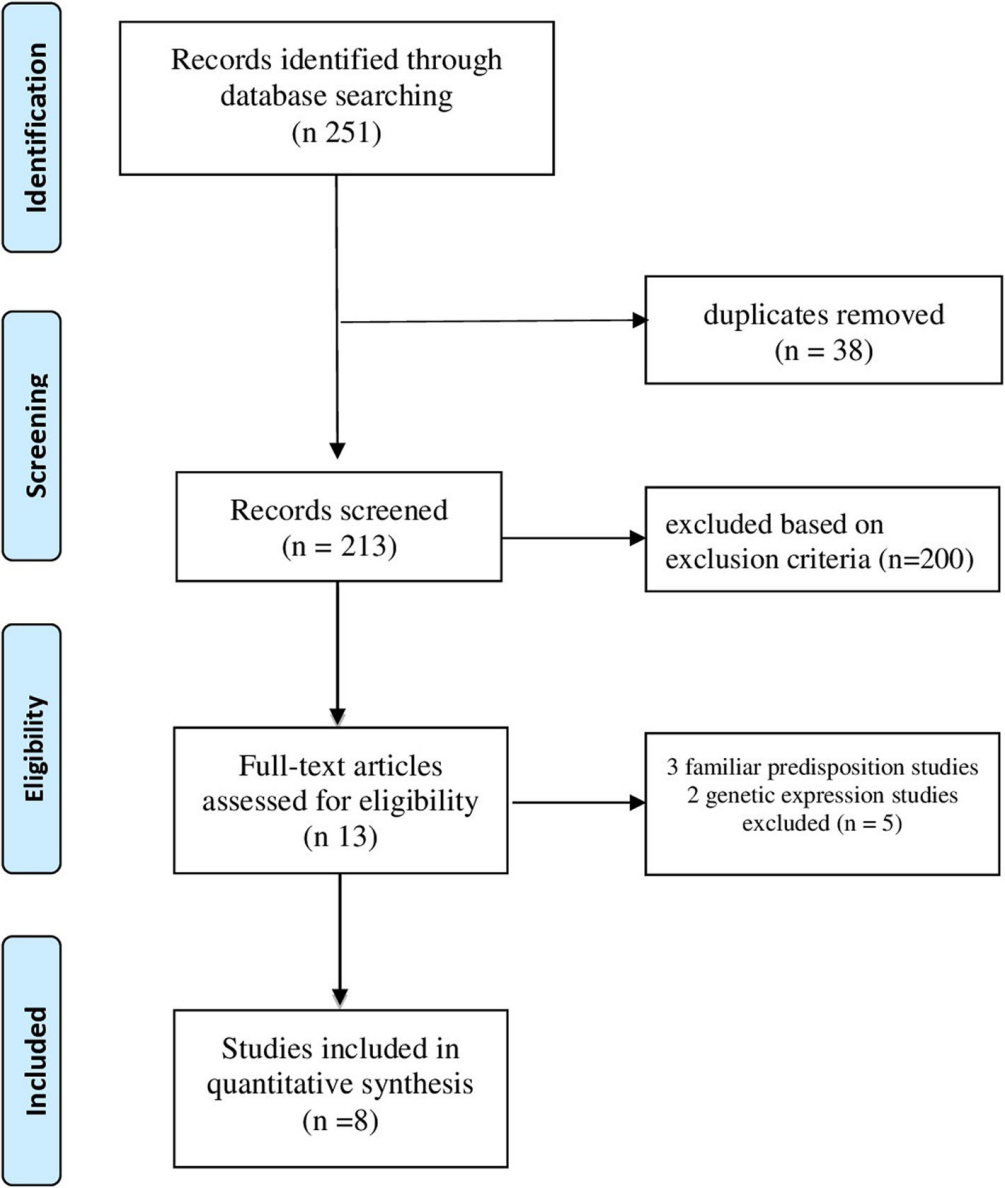
PRISMA 2009 Flow diagram

The literature search and the cross-referencing process resulted in 251 articles. 213 studies were assessed for relevance by title and abstract. 38 duplicates were removed and 200 were excluded because they did not meet inclusion criteria. After reading the remaining full-text articles, we rejected 3 studies about familial predisposition and 2 studies about genetic expression. Finally, 8 articles were included in the present review [19–26]. The flow-chart of literature search is shown in Fig. 1. Features of the studies are shown in Table 1.

**Table 1:** Study features

Author	Year	Type of Study	Cases	Controls	Diagnostic Criteria	Exclusion Criteria Case	Exclusion Criteria Control	Candidate Genes	Mean Age Group	Mean Age Control	Associated Genes
Teerlink et al. [19]	2015	Case-control on candidate genes	175	2595	MRI	Partial-thickness rotator cuff tear, tendinopathy only, significant arthritis, prior surgery	–	DEFB1, DENND2C, ESRRB, FGF3, FGF10, FGFR1			ESRRB (rs7157192)
Motta et al. [20]	2015	Case-control on candidate genes	203	207	Radiography and MRI	Older than 60 years and younger than 45 year, history of trauma, rheumatoid arthritis, autoimmune syndrome, pregnancy, and use of corticosteroids.	History of shoulder pain, impingement syndrome, presence of tendinopathy in other joints	DEFB1, DENND2C, ESRRB, FGF3, FGF10, FGFR1	51,8 (+/−5,1)	53,5 (+/− 5)	DEFB1, ESRRB (rs1676303), FGF3, FGF10, and FGFR1
Tashjian et al. [25]	2016	GWAS	311	2641	MRI	Partial-thickness rotator cuff tear, tendinopathy only, significant glenohumeral arthritis, prior surgery	–	GWAS	–	–	SAP30BP on chromosome 17 (*P* = 3.8E-9), SASH1 on chromosome 6 (*P* = 1.9E-7)
Assunção et al. [21]	2017	Case-control on candidate genes	64	64	MRI (case) ultrasound (control)	age > 65 years, traumatic tears	–	MMP-1, MMP-3	54 ± 6	53 ± 6	MMP-1, MMP-3 haplotype 2G/5A
Kluger et al. [22]	2017	case-control on candidate genes	155 (first cohort: 59; second cohort: 96)	76 (first cohort: 32; second, matched cohort 44)	Ultrasound	History of calcifying tendinitis, trauma or systemic disease/ inflammatory condition.	Prior operations of either shoulder, history of a humeral fracture or an infiltration or conservative shoulder treatment in the last 24 months or a systemic disease/inflammatory condition.	TNC, Col5A1, TIMP-1, MMP-1, MMP-2, MMP-3, MMP-9, and MMP-13	–	–	TNC
Roos et al. [26]	2017	GWAS	8357	94,622	–	–	Complete rupture of rotator cuff, Infraspinatus sprain, Rotator cuff sprain, Subscapularis sprain, Supraspinatus sprain, Repair of ruptured rotator cuff (acute), Repair of ruptured rotator cuff (chronic), Reconstruction of complete rotator cuff avulsion, Shoulder arthroscopy with rotator cuff repair	GWAS	–	–	rs71404070 (next to Cadherin8) (*P* = 2.31 × 10^− 8^)
Longo et al. [27]	2018	Case-control	93	206	MRI	Primary osteoarthritis of the operated or contralateral shoulder, inflammatory joint disease.	History of shoulder pain or rotator cuff pathology diagnosed by imaging or clinical examination	col5a1 rs12722	–	–	–
Salles et al. [28]	2018	Case-control	146	125	MRI	–	Tendinopathy history in any joint and who present no previous diagnosis of tendinopathy	FCRL3, FOXP3	26,93 +/− 6,03	21,62+/− 5,39	FCRL3

### Genetic variations

8 studies investigating genes variations associated with rotator cuff tears were included in this review. 6 studies were focused on candidate genes [19–24] and 2 studies were GWASs [25, 26].

The following candidate genes were investigated: *DEFB1, DENND2C, ESRRB, FGF3, FGF10, FGFR1, MMP-1, MMP-2, MMP-3, MMP-9, MMP-13, Col5A1, TNC, FOXP3, FCRL3*. A significant association between SNPs and rotator cuff disease was found for *DEFB1, FGFR1, FGFR3, ESRRB, FGF10, MMP-1, TNC, FCRL3*. Contradictory results were reported for *MMP-3* [21, 22]*.*

The two GWASs identified the following locus associated with rotator cuff tears: *SASH1*- rs12527089, *SAP30BP* - rs820218, rs71404070 located next to cadherin8 [25, 26].

### Quality assessment

The mean CMS score was 71 points (range from 71 to 84), indicating that the methodological quality of the included studies was good. There was no statistically significant difference among the CMS values of the examiners.

## Discussion

This systematic review outlines the current knowledge in the field of genetics in rotator cuff disease. Preliminary evidences of genetic and familiar predisposition to rotator cuff tears provided the basis for further studies that better highlight the importance of the genetic component in the pathogenesis of rotator cuff disease. In 2017, Dabija et al. [29] reviewed the literature on this topic describing the results of 4 studies investigating familiar predisposition and 3 studies investigating genes associated with rotator cuff tears. Up to day, we found other 5 studies focused on genetic variations associated with rotator cuff diseases. Even if the pathogenesis of rotator cuff disease is still largely unknown, recent studies on candidate genes and GWASs draw attention to SNPs associated with rotator cuff disease [27, 28, 30].

Several genes variations have been associated with rotator cuff tears. Interactions between genes, encoded proteins and environment play a complex role in the development of rotator cuff disease [35].

Motta et al. assessed 23 SNPs in 6 candidate genes (*DEFB1, DENND2C, ESRRB, FGF3, FGF10, and FGFR1*) in 203 cases and 207 controls [20]. The products of these genes are reported to have a role in tendon repair and degenerative processes. Rotator cuff disease was associated with certain haplotypes in *DEFB1, FGFR1, FGFR3*, and *ESRRB*. After adjustment by ethnic group and sex another association in *FGF10* was revealed.

The association of variants in *ESRRB* and rotator cuff disease was further demonstrated by Teerlink et al. [19]*.* They identified high-risk haplotypes in the *ESRRB* gene comparing genotypes of 175 patients with rotator cuff tears with 2595 genetically-matched Caucasian controls. *ESRRB* is a protein-encoding gene classified as an orphan nuclear receptor, since its exact ligand is not known. It is involved in hearing loss [31], stem cell pluripotency [32], and cellular remodeling of energy consumption under conditions of hypoxia [33]. *ESRRB* induces hypoxia-inducible factor (HIF) transcription, and their interaction may be involved in tenocyte apoptosis [34].

Several studies selected candidate genes on the basis of pre-existing association analyses for Achilles tendon ruptures (*TNC, Col5A1, and MMP-*3) [35–37], tendinopathies of the elbow (*Col5A1*) [38], ruptures of the posterior tibial tendon (*MMP-1*) [39] and matrix metalloproteinase genes *MMP-1, MMP-2, MMP-3, MMP-9, MMP-13, TIMP-1* that are specifically expressed in torn rotator cuff.

Kluger et al. found no differences in genotype and allele frequencies for SNPs in *MMP-1, MMP-2, MMP-3, MMP-9, MMP-13*, and *Col5A1* genes while six SNPs in Tenascin-C (*TNC*) were associated with degenerative rotator cuff tears [22]. Unlike their study, Assunção et al. [21] found a significant association between genetic polymorphism of *MMP-3* and rotator cuff tearing that may be explained by the smaller number of individuals evaluated, nonpairing between cases and controls for age, and known risk factors such as high blood pressure, and racial and genetic characteristics of the population. Moreover, Assunção et al. studied a different polymorphism in *MMP-1* (rs1799750) that was significantly associated with rotator cuff tearing.

In accordance with to Kluger et al. [22], no significant difference in allele and genotype frequencies of col5a1 was observed in the study by Longo et al. [23].

As demonstrated in in-vivo studies, the immune cells play a key role in the pathophysiology of rotator cuff tears. Salles et al. found an increased risk of tendinopathy associated with Fc receptor-like 3 polymorphism (*FCRL3 − 169 T > C*) [24]. *FCRL3* is a glycoprotein of the immunoglobulin receptor superfamily, expressed in Treg cells that may play a role as a negative regulator of Treg function [40–42].

GWASs are a powerful tool to pinpoint genes that may contribute to the risk of developing rotator cuff disease. The GWAS by Tashjian et al. identified two significant SNPs associated with rotator cuff tears: *SASH1* (rs12527089) and *SAP30BP* (rs820218) [25]. Those genes are associated with the cellular process of apoptosis. *SASH1* is a tumor suppressor gene that is ubiquitous expressed and, therefore, may be a potential candidate for dysregulation in musculoskeletal tissue. *SAP30BP* is a ubiquitously present transcriptional regulator protein on chromosome 17. It may act as a transcriptional co-repressor of a gene related to cell survival [43].

In another GWAS, Ross et al. found a SNP located next to cadherin8 significantly associated with rotator cuff injury. It encodes a protein involved in cell adhesion [26].

## Conclusion

Studies on candidate genes and GWASs identified several genes variation associated with rotator cuff tears, such as *DEFB1, FGFR1, FGFR3, ESRRB, FGF10, MMP-1, TNC, FCRL3, SASH1, SAP30BP*, rs71404070 located next to cadherin8.

Further investigations are warranted to identify complete genetic profile of rotator cuff disease and to clarify the complex interaction between genes, encoded proteins and environment. This may lead to individualized strategies for prevention and treatment of rotator cuff disease.

## Data Availability

The dataset supporting the conclusions of this article is included within the article.

## Abbreviations

CINAHL: Cumulative Index to Nursing and Allied Health Literature
CMS: Coleman Methodology Score
Col5A1: Collagen type V alpha 1 chain
DEFB1: b-defensin protein
DENND2C: DENN Domain Containing 2C
ESRRB: Estrogen-related receptor-b
FCRL3: FC-receptor-like 3
FGF: Fibroblast growth factors
FGFR: Fibroblast growth factors receptor
FOXP3: Forkhead box P3
GWASs: Genome-wide association studies
HIF: Hypoxia-inducible factor
MMP: Matrix metalloproteinases
PRISMA: Preferred Reporting Items for Systematic Reviews and Meta-Analyses
SAP30BP: SAP30-binding protein
SNP: Single-nucleotide polymorphism
TNC: Tenascin-C
